# Rice Putative Methyltransferase Gene *OsPMT16* Is Required for Pistil Development Involving Pectin Modification

**DOI:** 10.3389/fpls.2020.00475

**Published:** 2020-04-24

**Authors:** Kazuya Hasegawa, Shihomi Kamada, Shohei Takehara, Haruki Takeuchi, Atsuko Nakamura, Shinobu Satoh, Hiroaki Iwai

**Affiliations:** ^1^Graduate School of Life and Environmental Sciences, University of Tsukuba, Tsukuba, Japan; ^2^Faculty of Life and Environmental Sciences, University of Tsukuba, Tsukuba, Japan

**Keywords:** pectin, pectin methyltransferase, pectin methylesterification, pistil, rice (*Oryza sativa* L.), transmitting tissue

## Abstract

Pectin synthesis and modification are vital for plant development, although the underlying mechanisms are still not well understood. Furthermore, reports on the function of pectin in the pistil are limited. Herein, we report the functional characterization of the *OsPMT16* gene, which encodes a putative pectin methyltransferase (PMT) in rice. The cell walls of rice leaves contain less pectin, and chemical analysis of pectin in the flower organ had not been previously performed. Therefore, in the present study, the amount of pectin in the reproductive tissues of rice was investigated. Of the reproductive tissues, the pistil was especially rich in pectin; thus, we focused on the pistil. OsPMT16 expression was confirmed in the pistil, and effects of pectin methylesterification regulation on the reproductive stage were investigated by studying the phenotype of the T-DNA insertion mutant. The *ospmt16* mutant showed significantly reduced fertility. When the flowers were observed, tissue morphogenesis was abnormal in the pistil. Immunofluorescence staining by pectin-specific monoclonal antibodies of the pistil revealed that total pectin and esterified pectin were decreased among *ospmt16* mutants. These results indicate that *OsPMT16* contributes significantly to pistil development during reproductive growth.

## Introduction

Structurally and functionally, pectin is the most complex polysaccharide in the plant cell wall (Mohnen, 2008). Substantial evidence indicates that pectin plays essential roles in plant growth and development, which is consistent with the requirement for large numbers of genes for pectin synthesis and modification (Ridley et al., 2001; Mohnen, 2008; Atmodjo et al., 2013). Biochemically, pectins are polysaccharides that are rich in galacturonic acid (GalA); they are divided into three main types: homogalacturonan (HGA), rhamnogalacturonan-I, and rhamnogalacturonan-II (Willats et al., 2001). HGA biosynthesis and modification have recently been identified as key determinants of plant development (Wolf et al., 2009).

The cell wall plays important roles in plant development. It imposes spatial constraints on the plant cell; in higher plants, it provides mechanical strength, determines cell shape, participates in cell–cell communication, and protects against attacks by pathogens and predators (Somerville et al., 2004). Ordered deposition of cell wall materials and their composition is important for coordinating the planes of cell division and expansion during development (Baskin, 2001).

Plant cell walls are primarily composed of polysaccharides, including cellulose microfibrils and matrix components (McNeil et al., 1984; Gigli-Bisceglia et al., 2019). One matrix component is pectin, which is negatively charged and tends to form gel-like structures (Jarvis, 1984). Pectin and its modifications play important roles in plant physiological processes such as plant development and growth, leaf senescence, plant–pathogen interactions, and abiotic stress responses (Wolf et al., 2009; Lionetti et al., 2012; Palin and Geitmann, 2012; Fang et al., 2016; Qi et al., 2017).

Chemically, pectin is a mixture of heterogeneously branched polysaccharides (Ridley et al., 2001). The main pectic polysaccharide is linear HGA, which consists of linear α-1,4-linked d-GalA, a compound that is variably methyl esterified at C6 (Gigli-Bisceglia et al., 2019). Pectic polysaccharides are synthesized in the Golgi apparatus (Driouich et al., 1993; Calderan-Rodrigues et al., 2019), and a substantial portion of HGA is secreted in methyl esterified form (Li et al., 1997, 2002; Lennon and Lord, 2000). The degree of pectin methylesterification is important for determining the adhesive properties of pectin. The number of free carboxyl groups in pectin is determined by the degree of pectin methylesterification, which is regulated by the coordinated activities of pectin methyltransferases (PMTs), pectin methyl esterase (PME), and PME inhibitors (Senechal et al., 2014). Several PME genes have been identified and investigated (Tieman et al., 1992; Gaffe et al., 1997; Wen et al., 1999; Hongo et al., 2012). PMT activity has been described, and the enzyme properties characterized, in several plant species (Goubet et al., 1998; Goubet and Mohnen, 1999; Ishikawa et al., 2000; Łękawska-Andrinopoulou et al., 2013). However, functions of the PMT gene are not well understood, especially during the reproductive process.

Plant reproductive processes require active intercellular communication and cell-wall changes. Reproductive tissues are particularly rich in pectin compared with other tissues, indicating that pectin plays an important role in a variety of processes from pollination to fertilization (Lord, 2000; Mollet et al., 2000). During pollination, pollen recognizes stigma calcium and begins to germinate. After pollination, pollen tubes that enter the stigma extend through the adhering cells of the transmitting tissue toward the ovary (Lord, 2000). Consequently, dicotyledonous plants have been shown to contain a large amount of pectin in the transmission tissue along the pollen path (Iwai et al., 2006). However, reports on the amount of pectin and degree of methylesterification in the transmission tissues of monocotyledons are limited.

In *Arabidopsis thaliana*, defective mutations in the *TSD2* gene, which is presumed to encode a PMT, cause abnormal cell adhesion and hypocotyl differentiation during vegetative growth, and undifferentiated cells are observed. The problem is thought to arise during the PMT communication process (Krupkova et al., 2007). This gene is also expressed in the reproductive organs, indicating the importance of PMT in the reproductive process. However, to date, no phenotypes have been described for female reproductive tissues in PMT overexpression and deletion mutants.

To date, only a few studies have examined the role of pectin in female reproductive processes, and almost no reports have examined the relationship between these processes and the seed fertility. Therefore, in the present study, we focused on the pistil.

OsPMT16 showed most high levels of expression in pistil in the rice PMT genes according to the RiceXpro database. Hence, we focused on the OsPMT16 genes and T-DNA insertion mutant, and the effects of regulated pectin methylesterification on the reproductive stage was investigated by studying the phenotype. Since chemical analysis of floral pectin has not been previously performed, we also examined the amount of pectin in rice reproductive tissues.

## Materials and Methods

### Plant Material and Growth Conditions

Wild-type (WT) (cv. Dongjin) and *OsPMT16* (gene locus: Os06g0712800 (RAP-DB), AP014962 (GenBank) rice plants were cultivated in a greenhouse during the natural growing season.

### Extraction and Analysis of Cell Wall Polysaccharides

Alcohol-insoluble residue (AIR) generation, neutral sugar composition assay, uronic acid assays, and methyl ester assays were performed as described previously (Wood and Siddiqui, 1971; Filisetti-Cozzi and Carpita, 1991) with slight modifications.

Expanded leaf, glume, anther, and pistil were sampled for each of 4 to 8 independent biological replicates per treatment. Frozen samples were powdered with a mortar and pestle and washed in 80% EtOH. The supernatant was removed after centrifugation for 5 min at 15,000 × *g*. The pellet was washed with a mixture of methanol and chloroform (1:1 ratio) and then with acetone. A mixture of phenol, acetic acid, and water (2:1:1 ratio) was then added to the pellet. This process was repeated three times; the sample was then dried at room temperature for more than 1 h. After being washed with acetone, the samples were air-dried for more than 12 h. The alcohol-insoluble residues were used as the cell wall material. A total of 2 mg AIR was hydrolyzed with 2 M trifluoroacetic acid (TFA) at 121°C for 2 h. After hydrolysis, samples were centrifuged at 15,000 × *g* for 5 min. The supernatant was the TFA-soluble fraction. The pellets were hydrolyzed with 72% H_2_SO_4_ at room temperature for 2 h and then diluted to 4% H_2_SO_4_ and boiled for 1 h. H_2_SO_4_ solutions were neutralized with Ba(OH)_2_. In this study, pectin refers to the uronic acid content of AIR. Hemicellulose and cellulose are defined as the amount of neutral sugar in the TFA-soluble and TFA-insoluble fractions of cell wall material, respectively. Uronic acid was quantified with the sulfamate/carbazole method with 0.4 mg AIR (Filisetti-Cozzi and Carpita, 1991). Neutral sugar content in the TFA-soluble and TFA-insoluble fractions was determined with the phenol sulphuric acid method.

### DNA Extraction and Homo/Hetero Assay

Fully expanded mature leaves were sampled four independent biological replicates, frozen in liquid nitrogen and ground using a TissueLyser II instrument (Qiagen, Hilden, Germany). Total DNA was extracted using cetyltrimethylammonium bromide (CTAB) and amplified using forward (5′-TTTTCAGGACAAGCCTACCG-3′), reverse (5′-ATTGATCGGACAAGGACGAG-3′), and T-DNA (5′-ACAGGACGTAAC-3′) primers. The amplified DNA fragments were separated on 1% agarose gels, and bands were detected by staining with ethidium bromide.

### Vector Construction and Plant Transformation for Complementation Testing

For the complementation test, a 2,031 bp upstream sequence from a genomic DNA fragment containing the entire OsPMT16 gene was inserted into the binary vector pSTARA-R4, and pCAMBIA1300 was transformed as a control. The binary plasmids were introduced into *Agrobacterium tumefaciens* EHA105 by electroporation, and calli of the *ospmt16* mutant were transformed following the method of Hiei et al. (1994).

### RNA Extraction and Gene Expression Analysis

Plant material was frozen in liquid nitrogen and ground with a TissueLyser II instrument (Qiagen). Total RNA was extracted using the RNeasy Plant Mini Kit (Qiagen) and recombinant DNase I (Roche, Basel, Switzerland) according to the manufacturers’ protocols. Then, cDNA was synthesized using ReverTra Ace (Toyobo, Tokyo, Japan) according to the manufacturer’s protocol. The *OsPMT16* transcript level was quantified using forward (5′-GACCCGTTGTGATGATCTCC-3′) and reverse (5′-AATCTTGTGTTGGGGAGTGC-3′) primers. For the endogenous control, the 17S rRNA transcript was quantified using 17S rRNA-forward (5′-GCAAATTACCCAATCCTGAC-3′) and 17S rRNA-reverse (5′-CTATTGGAGCTGGAATTACC-3′) primers. The amplified DNA fragments were separated on a 1% agarose gel, and bands were detected by staining with ethidium bromide. Quantitative real-time polymerase chain reaction (qRT-PCR) was performed using TaqMan (Holland et al., 1991) or SYBR Green I (Qiagen) using cDNA as the template in a Model 7000 Sequence Detection System (Applied Biosystems, Foster City, CA, United States). To determine the role of *OsPMT16* during reproductive development, we used an *OsPMT16* promoter::GUS assay to monitor *OsPMT16* expression during reproductive development. A 2,031 bp 5′-UTR fragment of *OsPMT16* was amplified from the genomic DNA. The promoter fragment was then cloned into pBI121 for transformation into *Oryza sativa* (cv. Nipponbare) via *Agrobacterium* (Shu et al., 2015). GUS staining was performed on the flower as described by Shu et al. (2015). Briefly, samples were incubated in staining solution (20 mm X−Gluc in phosphate buffer) for 12 h at 37°C and then rinsed in 70% ethanol for microscopic observation.

### Immunohistochemistry

WT anthers at the same developmental stage were fixed with 4% PFA, 0.25% glutaraldehyde, and 0.05 M phosphate buffer (pH 7.5), and embedded in 5% agar. Sections (50-μm thickness) were cut using a Leica VT1200S microtome (Leica Microsystems, Wetzlar, Germany) and subjected to immunohistochemical analysis using the TSATM Kit #12 with horseradish peroxidase (HRP)-goat anti-rabbit IgG and Alexa Fluor 488 tyramide (Micro Probes/Invitrogen, Eugene, OR, United States) according to the manufacturer’s protocol. Primary LM19 and LM20 antibodies (PlantProbes, Leeds, United Kingdom) were used at a dilution level of 1:20. The HRP conjugate was used at a dilution level of 1:100. The negative control lacked the primary antibody. The sections were visualized using fluorescence microscopy (Leica Microsystems).

### Phylogenetic Analysis

We conducted a search of the SALAD database to find genes with the rice PMT domain pfam03141 (Methyltransf_29, Putative S-adenosyl-L-methionine-dependent methyltransferase). We identified 20 genes as candidates. Multiple sequence alignment was performed with ClustalX for full-length sequences of these rice candidate genes and *A. thaliana* (TSD2), followed by manual adjustment.

### Statistical Analysis

**FIGURE 1:**
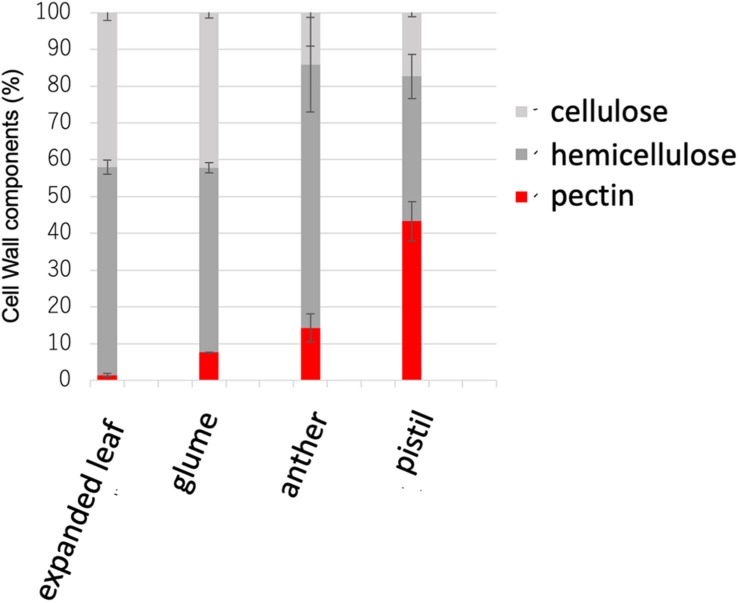
Cell wall sugar composition in the expanded leaf, glume, anther, and pistil. Pectin levels were measured as uronic acid content in the cell wall. Hemicellulose was measured as the amount of neutral sugar in the trifluoroacetic acid (TFA)–soluble fraction in the cell wall. Cellulose was measured as the amount of neutral sugar in the TFA-insoluble fraction in the cell wall. Data represents the means of independent biological replicates ± standard deviation (SD) for the expanded leaf (*n* = 7), glume (*n* = 8), anther (*n* = 4), and pistil (*n* = 4).

The data of Figures 1, 4–6 were expressed as the mean values ± standard deviations (SD) taken from 4 to 9 biological independent experiments. The experimental data of the samples were statistically analyzed through one-way analysis of variance (ANOVA) with Tukey’s *post-hoc* test using Statistica 13.1 software (StatSoft, Inc., Tulsa, OK, United States). The results with *p* ≤ 0.05 and *p* ≤ 0.01 were considered statistically significant.

## Results

### Cell Wall Composition Analysis of Rice Flowers

*Gramineae* plants including rice have been reported to contain low amounts of pectin in vegetative tissues (Vogel, 2008). However, no detailed analysis of pectin in the reproductive tissues of rice has been reported. Therefore, to investigate the cell-wall composition of the actual reproductive tissues of rice, Nipponbare WT mature leaves, flower buds before flowering, flower buds after flowering, and the cell walls of pistils were analyzed biochemically. The cell walls of vegetative tissues, such as mature leaves comprised only ~5% pectin, as previously reported (Sumiyoshi et al., 2015). However, in the reproductive tissues, the cell walls of pistils comprised ~43% pectin (Figure 1).

### Selection of Homozygotes for T-DNA Insertion Mutants and Complementary Testing

We searched the SALAD rice genome database for genes with rice PMT domains, and identified 20 genes as candidates. Amino acid sequences of the candidate genes, including the conserved domain of pfam03141 (Methyltransf_29, Putative S-adenosyl-L-methionine-dependent methyltransferase), were obtained from the RAP-DB rice annotation database. The amino acid sequence of the putative PMT gene *TSD2*, which was first reported in *A. thaliana*, was obtained from the *Arabidopsis* annotation database TAIR. A phylogenetic tree based on the sequences was created using multiple sequence alignment programs and ClustalX (Supplementary Figure S1). To date, few studies have examined the role of pectin in female reproductive development; therefore, we searched for rice PMT genes that were highly expressed in the pistil. According to the RiceXpro database, the OsPMT16 gene (Os06g0713800) had the highest expression. We then obtained the T-DNA insertion OsPMT16 mutant from the Pohang University of Science and Technology (POSTECH, Pohang, South Korea).

**FIGURE 2:**
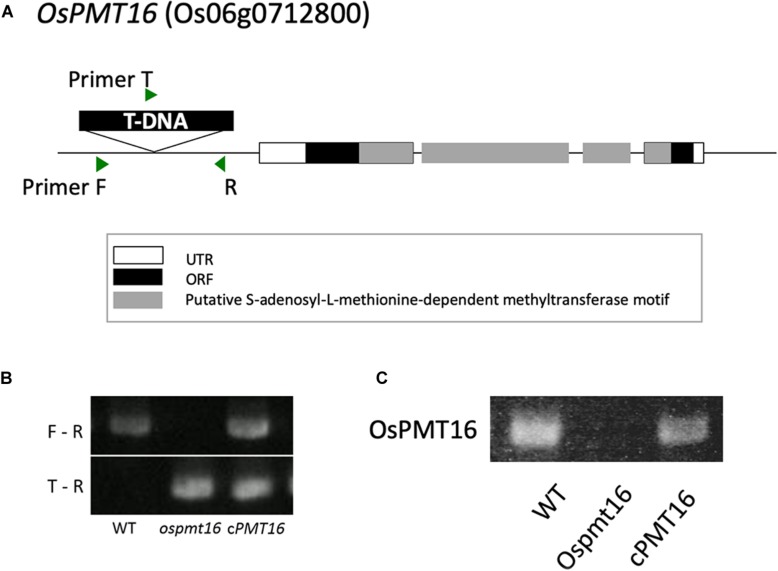
Structure of OsPMT16. The *ospmt16* homozygous line produced a null mutant. **(A)** Structure of *OsPMT16*, which contains a T-DNA sequence inserted at the promoter region. **(B)** Homo–hetero test of T1 mutants. F, R, and T indicate primer positions. **(C)** Results of quantitative reverse-transcription polymerase chain reaction (qRT-PCR) analysis of *OsPMT16* expression in flowers of the wild-type (WT), *ospmt16* mutant, and cPMT16 (complement).

The T1 generation was generated from ordered mutants and a homo/hetero assay was performed to select homozygous individuals using genomic PCR. In addition, complements (*cPMT16*) were prepared to identify the causative genes for phenotypic abnormalities observed in the mutants. A full-length genome construct including the original promoter region was inserted into the pSTARA-R4 vector and introduced into each mutant. T-DNA was inserted as a promoter at −272 bp in *OsPMT16* to create *ospmt16* mutants (Figure 2A). We confirmed that *OsPMT16* was not expressed in the mutant and that expression was restored in the complement (Figure 2C). Null-segregant individuals were isolated from *ospmt16* mutants and used for experiments as WT (Figure 2B).

### Gene Expression and Phenotypic Analysis of *ospmt16* Mutants During Vegetative Development

**FIGURE 3:**
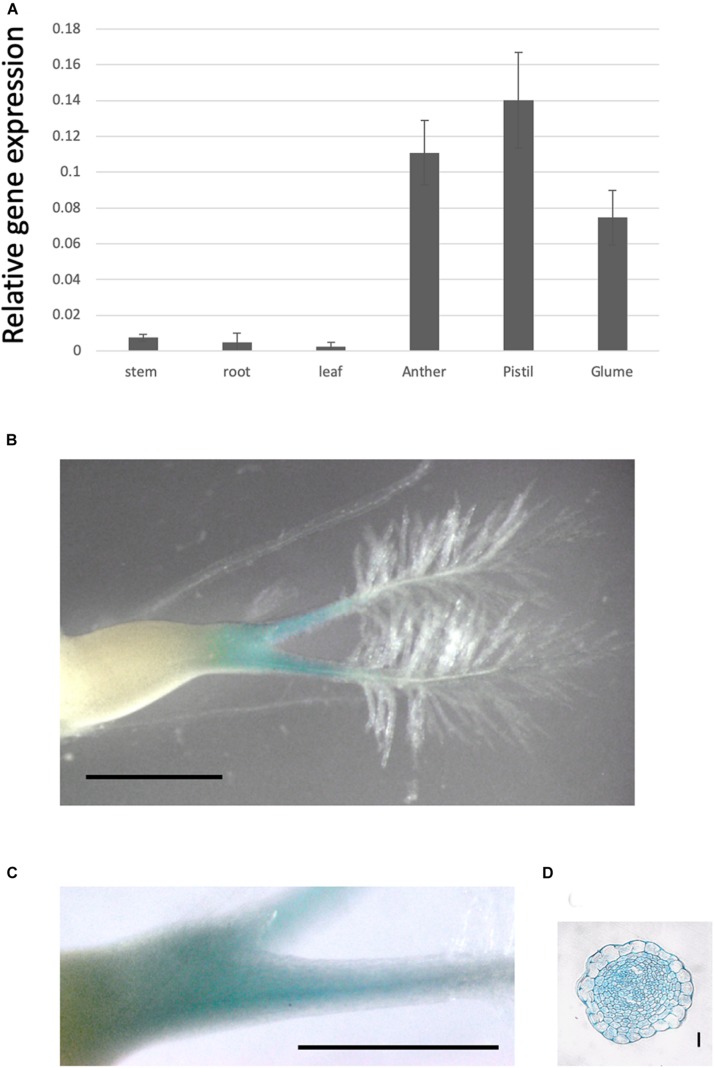
*OsPMT16* gene expression is high in pistil transmitting tissue. **(A)** Organ-specific expression patterns of *OsPMT16* in WT plants cultivated in an open field during the natural growing season. Leaves and stems at 60 days (mature plant) after seeding were used as vegetative stage organ samples. Bars represent means ± SD of four independent biological replicates. **(B)** Localization of pOsPMT16::GUS expression in the pistil. Bar, 1 mm. The experiments were performed at least twice with similar results. **(C)** Magnified image of a pOsPMT16::GUS pistil. Bar, 1 mm. **(D)** Cross-section of a pOsPMT16::GUS pistil. Bar, 10 μm.

To investigate gene expression in each tissue, we examined *OsPMT16* expression levels using qRT-PCR. The gene was strongly expressed in reproductive organs, especially the pistil and anther (Figure 3A); however, almost no expression was detected in the root, stem, or leaf. Particularly high GUS signal levels were observed in pistil transmitting tissues in OsPMT16promoter::GUS plants (Figures 3B–D).

**FIGURE 4:**
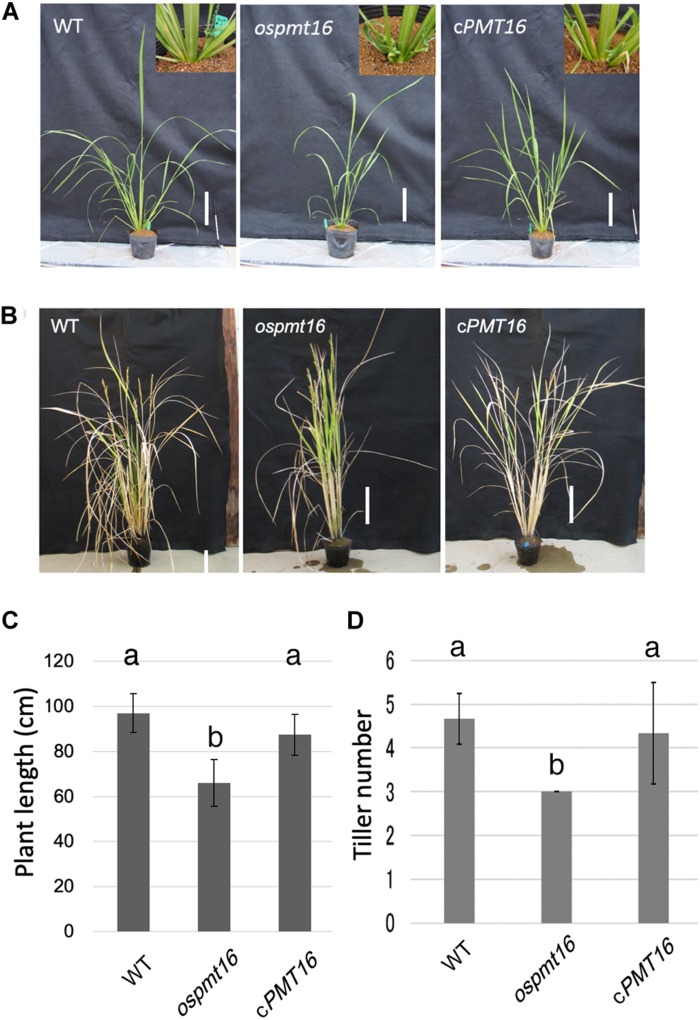
Phenotypes of WT, *ospmt16*, and cPMT16 plants during the vegetative stage. WT, *ospmt16*, and cPMT16 plant growth **(A)** 71 days after seeding and **(B)** after flowering. Bar, 10 cm. **(C)** WT, *ospmt16*, and cPMT16 plant length. Data represents the means of independent biological replicates ± SD for WT (*n* = 7), *ospmt16* (*n* = 8), and cPMT16 (*n* = 4). Compared with WT, *ospmt16* plants were shorter and **(D)** produced fewer tillers. Data represents the means of independent biological replicates ± SD for WT (*n* = 4), *ospmt16* (*n* = 9), and cPMT16 (*n* = 4). Bars with different letters indicate significant difference (*p* ≤ 0.05).

The phenotype of the *ospmt16* mutant during vegetative growth was investigated. The *ospmt16* mutant showed delayed vegetative growth compared with the WT (Figure 4C). The plant height on day 71 after sowing was short, and the number of tillers was small (Figures 4A,D). By the time of reproductive growth, the plant height was normal (Figure 4B), however, the number of tillers remained small (Figure 4).

### Disordered Pistil Morphology in the OsPMT16 Mutant

**FIGURE 5:**
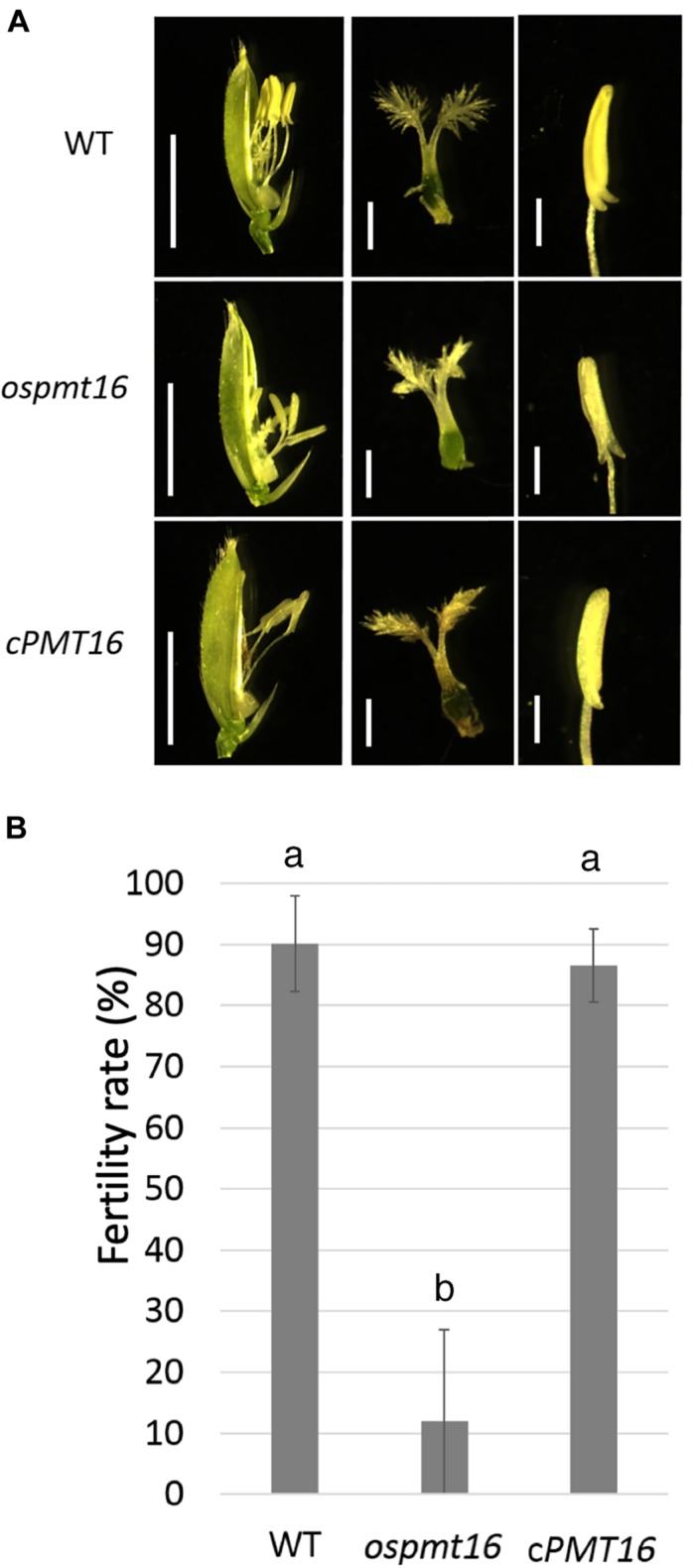
Phenotypic analysis of WT, *ospmt16*, and cPMT16 flowers. **(A)** Stamens of the *ospmt16* mutant had slightly smaller pistils and shorter anthers. Bars represent (left panels) 5 mm and (middle and right panels) 1 mm. In *cPMT16*, which was very similar to the WT, these phenotypes were not observed. **(B)** Seed fertility (proportion of normal seeds in all spikelets) represents the means of independent biological replicates ± SD for WT (*n* = 4), *ospmt16* (*n* = 9), and cPMT16 (*n* = 4). Despite very low fertility in the *ospmt16* mutant, only minor differences in reproductive organ morphogenesis were observed between *ospmt16* and WT. Bars with different letters indicate significant difference (*p* ≤ 0.01).

**FIGURE 6:**
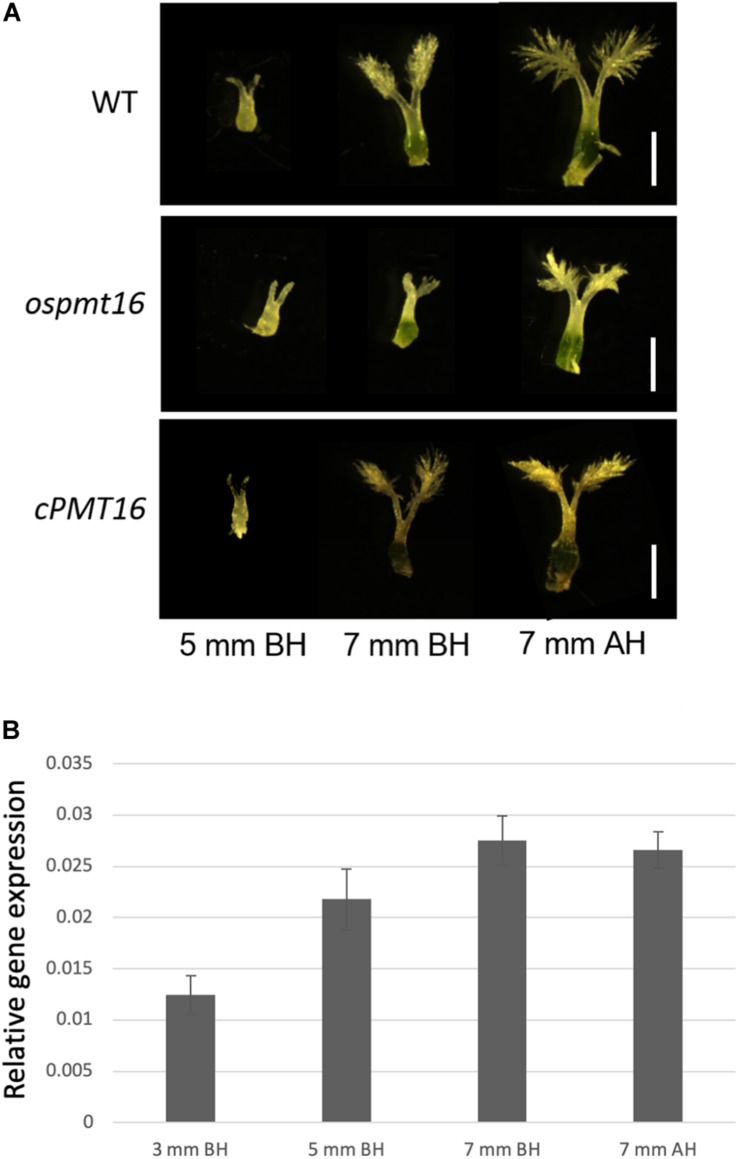
Phenotypic analysis of pistils of WT, *ospmt16*, and cPMT16 plants during flower development. **(A)** Pistil development was observed in flowers at glume lengths of 3, 5, and 7 mm before heading (BH) and 7 mm after heading (AH). Growth delays were observed in the stamen stigma and pistil during morphogenesis at 7 mm BH. **(B)** Pistil *OsPMT16* gene expression increased gradually during pistil growth.

During reproductive development, the fertility rate decreased by ~12% in the *ospmt16* mutant (Figure 5B). Flowers were sampled before heading and flowering, and their morphology was investigated. Pistils were slightly shorter in the ospmt16 mutant than in the WT, and the angle of the stigma opening was narrower. Stamens were slightly shorter in the *ospmt16* mutant (Figure 5A). In *cPMT16*, which was very similar to the WT, these phenotypes were not observed (Figure 5A). Despite the very low fertility of the *ospmt16* mutant, only minor differences in reproductive organ morphogenesis were observed between the *ospmt16* mutant and WT. To identify the stage in which abnormalities occurred during differentiation, the pistil was sampled at each stage and its morphology evaluated. Pistils were collected from flowers at glume lengths of 3, 5, and 7 mm before heading, and at 7 mm after heading. OsPMT16 gene expression increased gradually during pistil growth (Figure 6B). Delayed growth was observed in the stamen stigma and pistil during morphogenesis at 7 mm before heading (Figure 6A).

**FIGURE 7:**
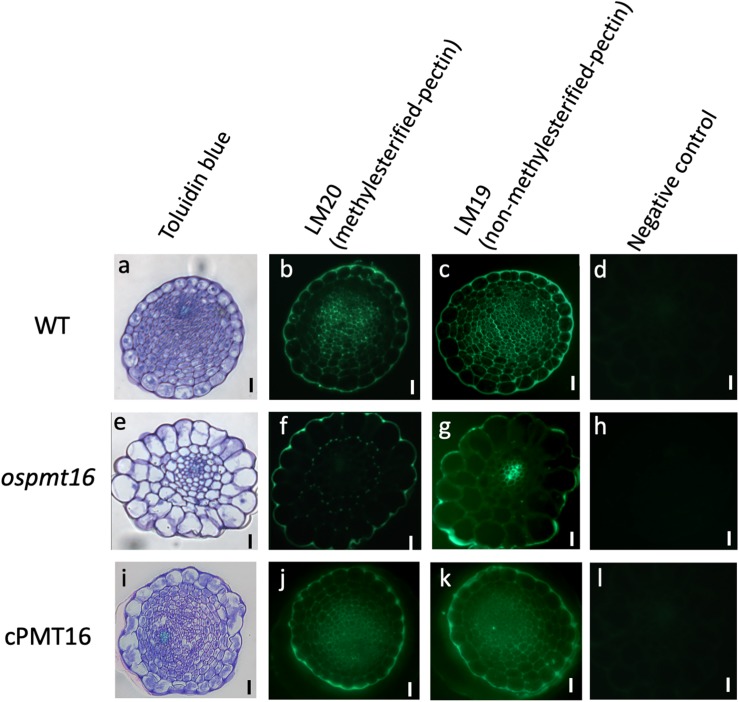
Immunohistochemistry of resin-embedded pistil sections at a glume length of 7 mm BH in WT, *ospmt16*, and cPMT16 plants. Cross-sections of **(a–d)** WT, **(e–h)** *ospmt16*, and **(i–l)** cPMT16 labeled with **(b,f,j)** anti-methyl esterified pectin (LM19) and **(s,b,k)** anti-esterified pectin (LM20) monoclonal antibodies. **(a,e,i)** Sections were stained by toluidine blue and observed under bright-field illumination. **(d,h,i)** Micrographs showing the negative control (without the first antibody step). All experiments were performed at least four times with similar results. Bars, 10 μm.

To identify the defects responsible for low fertility, we compared pistil cross-sections at 7 mm before the heading stage from OsPMT16 and WT plants. Toluidine blue staining indicated that the pistil of the same mutant OsPMT16 line exhibited abnormal morphogenesis compared to the WT (Figure 7). Cross-sections revealed that the WT pistil was composed of a single outer epidermis layer, transmitting tissues with high cell density, and a vascular bundle. However, in *ospmt16*, the outer epidermal cells and transmitting tissues of the pistil were vacuolated. Cells of transmitting tissues contained less cytosol, were disordered, and had far fewer cells than the WT. Normal vascular bundles were developed even in the *ospmt16* mutant. We examined cell wall sugar distribution using monoclonal antibodies against pectic polysaccharide epitopes. We prepared cross-sections by staining with de-methyl esterified pectin-specific antibody (LM19) and methyl esterified pectin-specific antibody (LM20). The *ospmt16* mutant exhibited decreased signals of both LM19 and LM20 in the pistil style. In WT, high LM20 signal levels were observed in pistil transmitting tissues; we also detected GUS signals in the transmitting tissues of pOsPMT16::GUS plants (Figure 3C). Slightly less uronic acid was detected in pistils of the *ospmt16* mutant than in the WT and cPMT16, but this difference was not significant (Supplementary Figure S2).

## Discussion

### In Rice Reproductive Tissues, Pistil Is Especially Rich in Pectin

Type I cell walls of dicotyledonous plants such as *A. thaliana* contain as much as 35% pectin in vegetative tissues, whereas rice type II cell walls contain approximately only 5% in the whole body (Yokoyama and Nishitani, 2004). However, an abnormal reproductive phenotype in pectin-modified rice has been reported (Jang et al., 2003; Sumiyoshi et al., 2015). In the present study, the constituent sugars in different organs of WT rice were investigated. Results revealed that mature leaves contained approximately only 5% pectin, which was consistent with reports in previous studies. However, when other organs were investigated, the cell walls in reproductive tissues, especially pistils, contained ~43% pectin (Figure 1). Based on these results, even in monocotyledonous plants known for having low amounts of pectin in vegetative tissues, pectin was shown to be abundant in reproductive tissues. Therefore, pectin likely plays an important role in the reproductive growth phase in rice as well as in dicotyledonous plants with reproductive tissues rich in pectin.

### Regulation of Pectin Methylesterification by *OsPMT16* Is Essential During Rice Vegetative Growth

PMT likely plays an important role in vegetative growth based on weak cell hypocotyl adhesion and abnormal leaf bud differentiation in *Arabidopsis* putative PMT gene *QUA2/TSD2* deletion mutants (Mouille et al., 2007; Krupkova et al., 2007). In the *ospmt16* mutant, an increase in plant height was delayed during vegetative growth and the number of tillers decreased (Figure 4). In the cell walls of rice tillers, the cellulose synthase GNT1 may be involved (Fanata et al., 2013), however, there are no reports on pectin. Based on the phenotypes observed in the present study, the degree of pectin methylesterification may control the number of tillers. However, the influence on growth is not substantial, and the plant can continue to develop to the flower stages. To date, reports of phenotypes of reproductive tissues of PMT-deficient mutants have not been published. Dicotyledonous plants such as *A. thaliana* are rich in pectin even in vegetative tissues. Therefore, abnormalities in the regulation of pectin methylesterification first affect vegetative growth, causing growth to cease and failure in flower development.

### *OsPMT16* Affects Pistil Morphogenesis

*QUA2/TSD2* is expressed in *Arabidopsis* flowers and in the double-deficient mutant of CGR2 and CGR3; the germination and elongation of pollen tubes weaken, the long-horned fruit becomes shorter, and the number of seeds decreases, indicating that PMT may play an important function during reproductive growth (Krupkova et al., 2007; Mouille et al., 2007; Kim et al., 2015). Furthermore, highly methyl esterified pectin may be involved in the morphogenesis of the style in the olive pistil (Suárez et al., 2013). In the present study, the *ospmt16* mutant was found to have significantly reduced fertility (Figure 5) and abnormal pistil and transmitting tissue morphogenesis (Figure 7). However, significant differences in phenotype morphogenesis were not observed in other organs (Figure 5). Therefore, pectin modification by OsPMT16 in transmitting tissues is required for normal pistil morphogenesis. PMT was considered to have an important function during the reproductive growth phase in rice with type II cell walls as well as in *A. thaliana* with type I cell walls.

Because levels of methyl esterified pectin, which was detected using the antibody LM20, was greatly reduced, the *ospmt16* mutant was hypothesized to exhibit decreased pectin methylesterification in the pistil style (Figure 7). In pistils that exhibited severe anomalies, signals of both LM19 (indicating dimethyl esterified pectin) and LM20 were greatly reduced (Figure 7). In the hypocotyl of the double-deficient mutant of CGR2 and CGR3, the total amount of uronic acid and methylesterification of uronic acid decreased (Kim et al., 2015). In the present study, the amount of uronic acid, which is the main component of pectin, in pistils differed little between *ospmt16* and WT (Supplementary Figure S2). Therefore, the amount of pectin does not have noticeable effects on the *ospmat16* phenotype. In addition to controlling the degree of methylesterification, *OsPMT16* may be involved in the biosynthesis and secretion of pectin. In the *ospmt16* mutant, anthers were abnormal at every stage from the very beginning of morphogenesis and shorter than WT anthers (Figure 5). In addition, we observed a pollen decrease of about 20%, as well as abnormal anther and pollen formation (Figure 1). Therefore, pectin methylesterification by *OsPMT16* is not essential for pollen maturation. In addition, the pectin originally synthesized at the tip of the pollen tube is highly methyl esterified (Lennon and Lord, 2000). Reduced amounts of methyl esterified pectin at the germination site of the tube have been reported (Kim et al., 2015). Therefore, pollen tube elongation may be affected by the *OsPMT16* mutation.

QUA2/TSD2, which is presumed to be an *Arabidopsis* PMT, has an S-adenosyl-L-methionine binding domain (SAM domain, pfam03141: Methyltransf_29), and GUS staining has been reported in reproductive tissues such as the pistil (Krupkova et al., 2007). In the present study, the amino acid sequence of OsPMT16 was also found to have an SAM domain. The OsPMT-16 gene sequence had 51% homology to QUA2/TSD2. In the phylogenetic tree, OsPMT16 and QUA2/TSD2 formed different clusters (Supplementary Figure S1). These two genes are highly likely to encode PMTs because pectin methylesterification in pistils decreased in the *ospmt16* mutant, and the degree of pectin methylesterification was restored in the complement. When cells differentiate, they need to communicate closely with surrounding cells (Kepinski, 2006). In the QUA2/TSD2 deletion mutant, abnormalities in cell adhesion in the hypocotyl occurred, indicating the possibility of an abnormality in intercellular communication (Qu et al., 2016; Xu et al., 2017). In the present study, the complement test confirmed the recovery of abnormal morphology. The results showed that pectin methylesterification was not regulated normally in the *ospmt16* mutant, and insufficient adhesion caused by pectin defects resulted in abnormal cell-to-cell communication and differentiation. *OsPMT16* contributes significantly to the development and maturation of the pistil for reproductive growth.

## Data Availability Statement

The datasets generated for this study are available on request to the corresponding author.

## Author Contributions

HI supervised the project in conception and execution. KH, SK, ST, HT, AN, and HI carried out the experiments. KH, SK, and HI analyzed and interpreted the data. KH and HI wrote the main manuscript text. KH, AN, SS, and HI revised the manuscript and participated in discussions of the research.

## Conflict of Interest

The authors declare that the research was conducted in the absence of any commercial or financial relationships that could be construed as a potential conflict of interest.

## Abbreviations

PMT: pectin methyltransferase
PME: pectin methyl esterase
WT: wild-type.
