# Supplementary material for: Rice Putative Methyltransferase Gene OsPMT16 Is Required for Pistil Development Involving Pectin Modification
Source: Front Plant Sci. 2020 Apr 24;11:475. doi: 10.3389/fpls.2020.00475 (PMC7212358; doi:10.3389/fpls.2020.00475)
Supplement: Supplementary file 1 [file Data_Sheet_1.PDF]

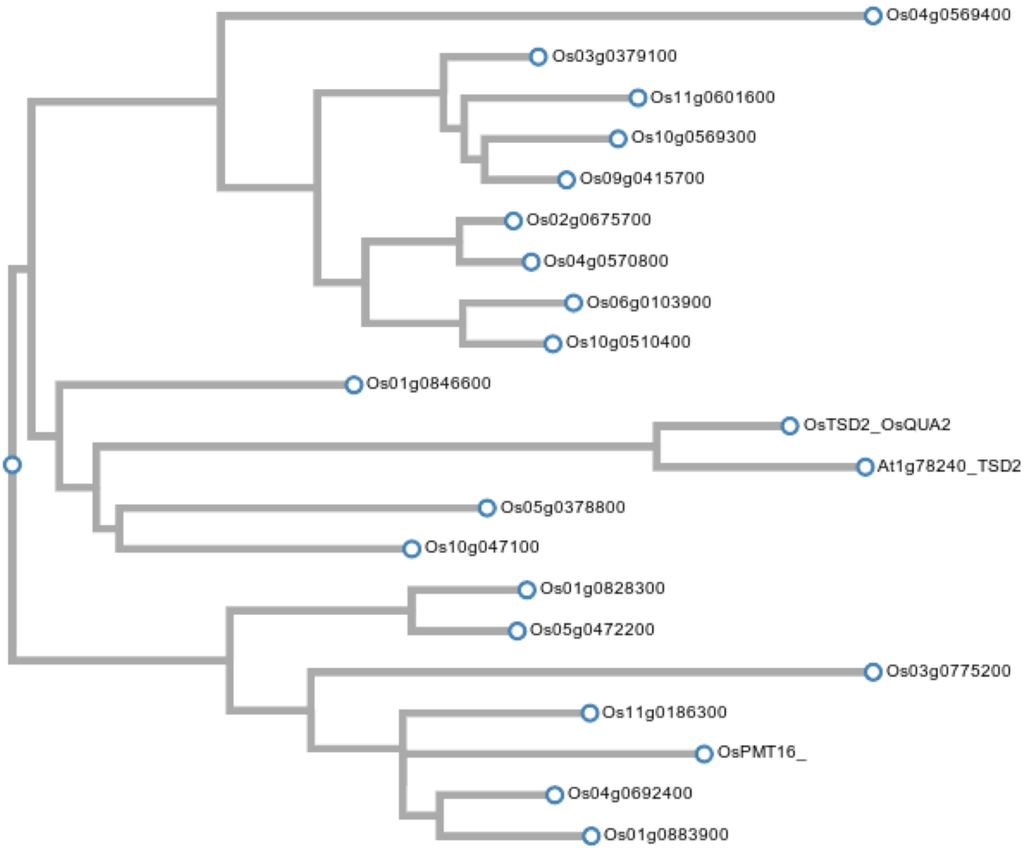

Figure S1. Phylogenetic tree of putative pectin methyltransferase (PMT) genes in *Oryza sativa* and *Arabidopsis thaliana* (*TSD2*). Multiple sequence alignment of protein sequences was performed to construct the tree using ClustalX.
