# Supplementary material for: Rice Putative Methyltransferase Gene OsPMT16 Is Required for Pistil Development Involving Pectin Modification
Source: Front Plant Sci. 2020 Apr 24;11:475. doi: 10.3389/fpls.2020.00475 (PMC7212358; doi:10.3389/fpls.2020.00475)
Supplement: Supplementary file 2 [file Data_Sheet_2.PDF]

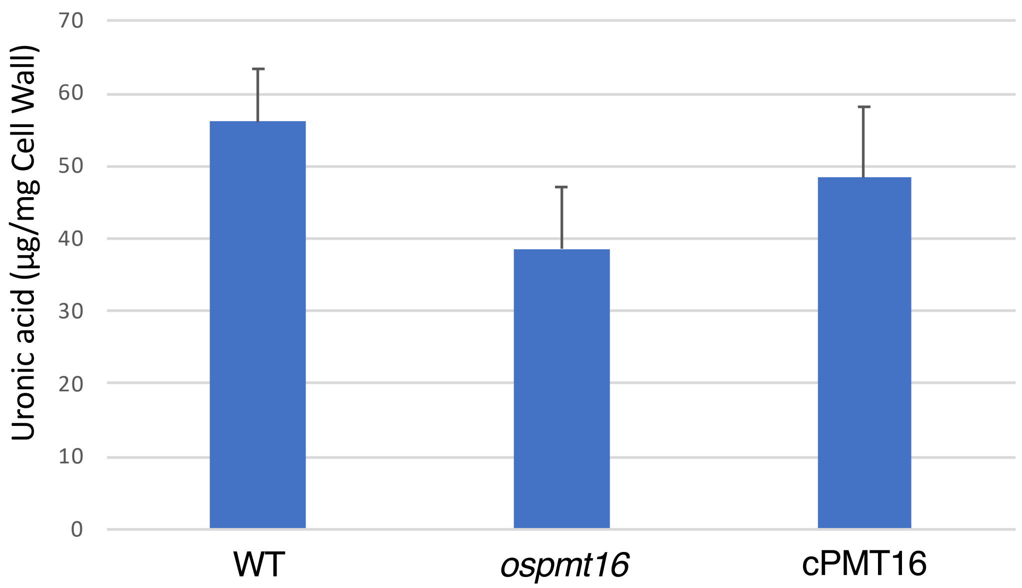

Figure S2. Amount of uronic acid in the pistil cell wall in wild-type (WT), *ospmt16*, and cPMT16 plants. Uronic acid content was measured in alcohol-insoluble residue (AIR) of the pistil. Bars indicate standard deviation (SD) (n = 4).
